# Preliminary Screening of Soils Natural Radioactivity and Metal(loid) Content in a Decommissioned Rare Earth Elements Processing Plant, Guangdong, China

**DOI:** 10.3390/ijerph192114566

**Published:** 2022-11-06

**Authors:** Yaole Huang, Wangfeng Wen, Juan Liu, Xiaoliang Liang, Wenhuan Yuan, Qi’en Ouyang, Siyu Liu, Cem Gok, Jin Wang, Gang Song

**Affiliations:** 1 Guangdong Provincial Key Laboratory of Radionuclides Pollution Control and Resources, School of Environmental Science and Engineering, Guangzhou University, Guangzhou 510006, China; 2 CAS Key Laboratory of Mineralogy and Metallogeny/Guangdong Provincial Key Laboratory of Mineral Physics and Materials, Guangzhou Institute of Geochemistry, Chinese Academy of Sciences, Guangzhou 510640, China; 3 Department of Metallurgical and Materials Engineering, Faculty of Technology, Pamukkale University, Denizli 20160, Turkey

**Keywords:** decommission, γ radiation air-absorbed dose rate, natural radioactivity, metal(loid) pollution

## Abstract

Radiological aspects such as natural radioactivity of ^238^U, ^232^Th, ^226^Ra, ^40^K combined with potentially toxic metal(loid) (PTM) distribution features were seldom simultaneously investigated in rare earth element (REE) processing activities. This work was designed to investigate the distribution levels of natural radioactivity, air-absorbed dose rate of γ radiation as well as PTMs at a typical REE plant in Guangdong, China. Ambient soils around REE processing facilities were sampled, measured and assessed. The natural radioactivity of radionuclides of the samples was determined using a high-purity germanium γ-energy spectrometer while the air-absorbed dose rate of γ radiation was measured at a height of 1 m above the ground using a portable radiometric detector. The PTM content was measured by inductively coupled plasma mass spectrometry (ICP-MS). The results showed that the specific activities of the radionuclides ranged from 80.8 to 1990.2, 68.2 to 6935.0, 78.4 to 14,372.4, and 625.4 to 2698.4 Bq·kg^−1^ for ^238^U, ^226^Ra, ^232^Th, and ^40^K, respectively, representing overwhelmingly higher activity concentrations than worldwide soil average natural radioactivity. The radium equivalent activity and external hazard index of most samples exceeded the limits of 370 Bq·kg^−1^ and 1, respectively. The measured air-absorbed dose rate of γ radiation was in a range of 113~4004 nGy·h^−1^, with most sites displaying comparatively higher values than that from some other REE-associated industrial sites referenced. The content levels of PTMs of Cu, Ni, Zn, Mn, Pb, Cd, Cr, and As were 0.7~37.2, 1.8~16.9, 20.4~2070.5, 39.4~431.3, 2.3~1411.5, 0.1~0.7, 6.7~526.1, and 59.5~263.8 mg·kg^−1^, respectively. It is important to note that the PTM contents in the studied soil samples were 2.1~5.4 times higher for Zn-As and 1.4 times higher for Pb than the third level of the China soil standard while 2.5~13 times higher for Zn-As and 1.2 times higher for Pb than Canadian industry standard. The findings call for subsequent site remediation to secure the ecological environment and human health after the REE processing plant was decommissioned.

## 1. Introduction

Rare earth elements (REEs) are widely applied in high-technology fields and various clean energy products due to their unique physical and chemical properties [1,2]. They comprise fifteen elements of the lanthanum series and yttrium and can be present in more than 250 different minerals [3,4]. However, the exploitation and processing of REE-containing minerals may cause various environmental issues due to the fact that these special minerals usually bear both natural radionuclides (such as ^238^U, ^226^Ra, ^232^Th, and ^40^K) that may pose gamma radiation or other kinds of radiation hazard, and potentially toxic metal(loid)s (PTMs such as cadmium, arsenic, lead, and zinc) [1]. Radionuclides and PTMs can be transferred from the REE minerals to the environmental matrix via natural process such as air transportation, rain leaching, and surface run off, or by anthropogenic REE processing activities such as chemical leaching and extraction, generating large number of processing residues in the form of airborne particles, solid wastes, and wastewater. These processing wastes may transfer to the soil–plants system, and finally to human beings through food chain. Long-term exposure to radioactivity or radionuclides may induce many health problems such as acute leukemia, anemia, necrosis of the mouth, tooth fracture, cataracts, and even cancers of the lung, hepar, bone, and kidney [5].

On the other hand, the processing of REE minerals may also result in and increase the occurrence of PTM contamination in the environment. It is reported that the irregular disposal of mining tailings and processing slags has been considered to be one of the principal causes for PTM contamination in the soils near REE smelter [6]. Even though the REE smelting activities have ceased, large amounts of waste residues may continue to be weathered, the dusts bound with considerable amount of PTMs from the above-mentioned wastes can be drifted and settled to long distance, further leading to the accumulated level of PTMs in ambient soils [7]. PTMs can appear enriched in organisms by migration and/or transformation combined with food chain when they are present in excessive concentrations, thus inevitably threatening the ecological safety of the surrounding environment and the public health of residents [8,9].

Since China has become the biggest producer of the REEs resource [10], the mining and processing of REE minerals inevitably induce a variety of environmental issues. For example, enriched REEs were also found in surface soils to varying degrees (total content of REEs ranged from 156 to 56,500  mg·kg^−1^ with an average value of 4670  mg·kg^−1^), influenced by the mining of REE minerals, around the Bayan Obo deposit in China [11]. The study also showed that the scale of REE mining had experienced a rapid expansion during the 1988–2010 period with a total mined area of about 66.29 km^2^ in the southern Jiangxi province of China [12]. According to the statistics of Xinfeng county in Jiangxi province, the area of farmland which was covered by REE tailings had reached 286 hm^2^, turning into a sandy desert and eventually not suitable for cultivation [13]. In India, the mining and processing of REE minerals along the coastline of Chavara has led to the geo-environmental degradation of the surrounding area within a radius of 1600 hm^2^ [14]. Increased environmental issues induced by REE mining and processing are threatening the ecological environment and living organisms; therefore, the monitoring and investigations of these related sites are of great necessity [15,16]. It is important to highlight that the studies on natural radioactivity and related doses exposed in the working area after the REE facilities are decommissioned were rarely reported.

Hence, the objectives of this study were to (i) investigate and evaluate the environmental air-absorbed dose rate of γ radiation and (ii) determine the specific activity of radionuclides of ^238^U, ^232^Th, ^226^Ra, ^40^K, and the content level of PTMs in ambient soils inside a typical decommissioned REE plant. The study would help to understand the radioactivity level and the pollution status of PTMs in REE processing industry, as well as provide an essential reference for the subsequent remediation and/or disposal of affected soils. The findings would also provide important data for the comparison of radionuclides-containing mineral ores or tailings in other nations.

## 2. Materials and Methods

### 2.1. Site information and Sample Collection and Treatment

**Figure 1:**
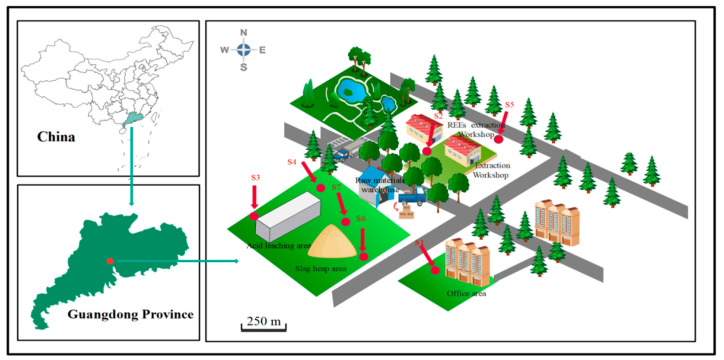
Schematic illustration of sampling sites in a decommissioned REE plant, Guangdong, China.

The decommissioned REE plant was located in Guangdong Province, China. Soil samples were collected near the following sites: office area (S1), REE extraction area (S2, S5), grassland close to acid leaching area (S3, S4), and slag heap area (S6, S7), as shown in Figure 1. Three samples were collected from each site and each individual soil sample kept a weight of ~1 kg after removal of biological debris, stone and being mixed homogeneously, then immediately placed in polythene zip lock bags prior to laboratory analysis. All the samples were dried in an oven at 105 ± 5 °C until the constant weight was obtained, followed by being ground to grain size of less than 0.25 mm, and then placed and sealed in the cylindrical PVC containers to prevent the escape of radioactive gases of ^220^Rn and ^222^Rn and kept impermeable for around 30 days to assure the equilibrium between ^238^U, ^232^Th, and related decay progenies [2]. It is worth to note that actual arsenic content might be higher than the values reported owing to the relatively higher drying and digestion temperature.

### 2.2. Measurement of Air-Absorbed Dose Rate of γ Radiation and Specific Radioactivity

The air-absorbed dose rate of γ radiation in the sampling area was measured in situ using a portable radiometric instrument detector (Inspector alert) at the height of 1 m above the ground. The soil radioactivity of ^238^U, ^226^Ra, ^232^Th, and ^40^K was measured with a low background Canberra high-purity Ge detector (HP Ge), with a resolution of 1.8 keV at 1332 keV gamma-ray line of ^60^Co source. Measurement data were obtained and analyzed by DSA-1000 spectrum analyzer (Canberra, USA) and GENIE-2000 software. The detailed measurement rationale of ^238^U, ^226^Ra, ^232^Th and ^40^K, and the calibration, procedure, measurement time were referred to [2]. The minimum detection limits were 0.2−0.4 Bq kg^−1^ for ^238^U, ^226^Ra, and ^232^Th, while the were 1.6 Bq kg^−1^ for ^40^K.

### 2.3. Radiation Exposure Assessment

The most common radiation exposure assessment included radium equivalent activity *R*_activity_, external radiation index (*I*_r_), and annual effective dose rate (*D*_aedr_) [17]. *R*_activity_ is a parameter suggesting the effective activities of ^226^Ra, ^232^Th and ^40^K in a single quantity, considering the radiation hazards related to them [2]. *I*_r_ was assumed to be lower than 1 to assure the radiation hazard from gamma radiation is negligible while attention should be paid when it is larger than 1 [18]. *D*_aedr_ was used to estimate the health effects on the public exposed to radiation in terms of fatal cancers occurring per sievert (Sv) [19]. The calculation formulas of *R*_activity_, *I*_r_, and *D*_aedr_ are as follows:

(1) $$R_{\mathrm{activity}}{=C}_{\mathrm{Ra}}+1{.43C}_{\mathrm{Th}}+0{.077C}_{K},$$

(2) Ir=CRa370+CTh259+CK4810,

(3) $$D_{\mathrm{aedr}}=D\times 8760\times 0.2\times 0{.7\times 10}^{-3},$$

where $C_{\mathrm{Ra}}$, *C*_Th_,${\;\mathrm{and}\;C}_{K}$ refer to the specific activity of ^226^Ra ^232^Th, and ^40^K, respectively. *D* and 8760 are the measured outdoor absorbed gamma dose rate (nGy·h^−1^) and 8760 h of one year, 0.2 is the outdoor residence factor, 0.7 (Sv Gy^−1^) is the conversion coefficient of absorbed dose in the air to the effective dose received by the public.

### 2.4. Determination of PMTs Content and Risk Assessment

About 0.1 g soil samples were accurately weighed and placed into a 100 mL polytetrafluoroethylene (PTFE) container together with 5 mL of 68% (*v*/*v*) HNO_3_, 3 mL of 40% (*v*/*v*) HF, and 1 mL of 30% (*v*/*v*) H_2_O_2_ and heated on an electric hot plate at 180 °C until near dryness. The digestion was subjected to repetition until a clear solution was obtained, followed by further heating until dryness to get rid of HF. Finally, the solution was diluted to 100 mL with deionized water (Milli-Q Millipore, 18.25 MΩ/cm) prior to equipment measurement of Pb, As, Cr, Mn, Ni, Cu, Zn, and Cd using inductively coupled plasma mass spectrometry (ICP-MS, PE–Sciex Elan 6100 DRC–II, PerkinElmer, Waltham, MA, USA). The certified standard reference materials of OREAS were applied and assured for the quality control, which displayed good consistency with reference values (Table 1). 

The single factor pollution index (*P*_m_) and comprehensive pollution index (*P*) are widely adopted when assessing the PTM pollution status in the soils [20]. Their definition is shown in Formulas (4) and (5). *P*_m_ ≤1 indicates no pollution; 1 < *P*_m_ ≤ 2 indicates slight pollution; 2 < *P*_m_ ≤ 3 indicates moderate pollution, and *P*_m_ > 3 indicates heavy pollution.

(4) Pm=CmSm,

(5) $$P=\sqrt{\frac{{\left(\frac{1}{n}\sum_{i=1}^{n}P_{i}\right)}^{2}+[\max\left(P_{i}\right){]}^{2}}{2}},$$

where $C_{m}$ is the actual measured value of the single PTM in soils; $S_{m}$ is the evaluation criteria of each PTM in background soils of Guangdong, China according to Cheng et al. [21]; $P_{i}$ and max ($P_{i}$) are each individual value and maximum value of single factor index of PTMs in soils; n is the total number of PTM species.

### 2.5. Statistical Analysis

Correlation analysis is a typical statistical approach to quantitatively describe and analyze the degree of correlation between two or more variables. To study the correlation among ^238^U, ^226^Ra, ^232^Th, ^40^K and among the elements of PTMs, it is suitable to apply correlation analysis to explore the correlationship between concerned radionuclides or PTMs in soils using the Origin software (version 9.7).

### 2.6. Soil Mineralogical Composition Characterizations

Semi-quantitative X-ray diffraction (XRD) was applied to analyze selected soil samples (S1, S2, S5, S6, and S7) mineral compositions. The identification of certain minerals was applied with MDI Jade 5.0 equipped with the JCPDS PDF-2 database [22]. In addition, selected soils (S2 and S5) were scanned by Transmission Electron Microscope (TEM) equipped with a High-Resolution Transmission Electron Microscope (HRTEM, 200 kV), which displays the elemental distribution and mineralogy of the studied soils at the nano/micro-scale. The mineralization and composition of the particles from the soils were also investigated by the Fast Fourier Transformation (FFT) image electron microscope (EM) maps [23].

## 3. Results and Discussion

### 3.1. Radioactivity Level of Surveyed Soils 

**Figure 2:**
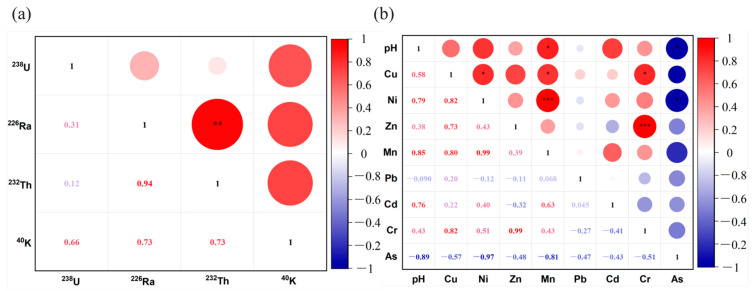
(**a**) Correlation analysis between ^238^U, ^226^Ra, ^232^Th, and ^40^K. (**b**) Correlation analysis between pH and 8 PTMs. Significance level: * *p* < 0.05, ** *p* < 0.01, *** *p* < 0.001.

The measured activity concentrations of natural radionuclides ^238^U, ^226^Ra, ^232^Th, and ^40^K in soil samples in different locations are displayed in Table 2. The activity concentrations of ^238^U ranged from 80.8 to 1990.2 Bq kg^−1^ with arithmetic mean of 518.9 Bq kg^−1^, which was 13 times the world average activity (40 Bq kg^−1^). The highest ^238^U activity concentration was found in REE extraction area (S2). ^226^Ra ^232^Th, and ^40^K also had high radioactivity level, which were 73.3, 127.3, and 3.3 times the world average soil activity (35, 30, and 400 Bq kg^−1^), respectively. The highest activity concentration of ^226^Ra, ^232^Th, and ^40^K was found in site of S5, S4 and S5, respectively. Through the correlation analysis (Figure 2a), significant correlations were observed between different radionuclides of the REE plant. All four radionuclides showed high positive correlations, and the correlation coefficients of ^226^Ra and ^232^Th were as high as 0.94, with a high degree of similarity, suggesting that ^26^Ra and ^232^Th may have similar sources.

This indicated that raw materials or waste slags were likely to be spilled out in the REE extraction or transportation process since the soils close to the REE extraction area were found with some slag-like matters, causing the radionuclides to gradually migrate to the ambient soils. Therefore, it is necessary to treat the waste residue and remediate the soils near the REE extraction area before the decommissioned REE plant could be used for any other purpose.

### 3.2. Air-Absorbed Dose Rate of γ Radiation

The in situ measurement results of γ radiation air-absorbed dose rates are shown in Table 3. Evidently, the levels of γ radiation air-absorbed dose rates in the study area varied significantly between 113 and 4004 nGy h^−1^ and followed in sequence: S1 < S3 < S6 < S7 < S2 < S4 < S5. It is important to note that most of the measured results of γ-ray air-absorbed dose rate, especially for S4 and S5, were significantly higher than those measured near other samples. As shown in Table 3, the average dose rate of the studied REE plant sampling sites was found to be remarkably higher than the value of worldwide average in outdoor environment of 59 nGy∙h^−1^. In addition, it is notable that these measured levels were comparable with or even higher than similar REE sites or other types of metal mining/tailings sites (Table 3), thus marking the surveyed sites as a particularly high natural radiation area. The calculated radiation exposure values of *R*_activity_, *I*_r_, and *D*_aedr_ are shown in Table 4. The results showed that both the *R*_activity_ and *I*_r_ of most sampling sites significantly exceeded the recommended limit values (*R*_activity_ limit value was 370 Bq·kg^−1^ while *I*_r_ was 1 [25,26,27]). Specifically, both the *R*_activity_ and *I*_r_ of S1, S2, S4, S5, and S7 were approximately 1.9, 22, 50, 75, and 3.3 times the limit value, respectively. Only the values of S3 and S6 were below the recommended limit level, indicating that the ambient soils and waste residues in the surveyed REE plant had a high risk of radiation hazards. However, in terms of annual effective dose rate, the global average *D_aedr_* listed by UNSCEAR report was 480 μSv·a^−1^ [28].

Comparatively, the *D*_aedr_ values of S1, S3, S6, and S7 fell roughly within the average *D*_aedr_ by UNSCEAR, implying that the annual external gamma radiation in some areas inside REE plant can be accepted. However, S2, S4, and S5 significantly exceeded 480 μSv·a^−1^, which was higher than a factor of 2–5, implying a non-negligible accumulated risk of gamma radiation from some areas inside the decommissioned REE plant.

### 3.3. Pollution Evaluation on the Basis of PMTs Content

**Figure 3:**
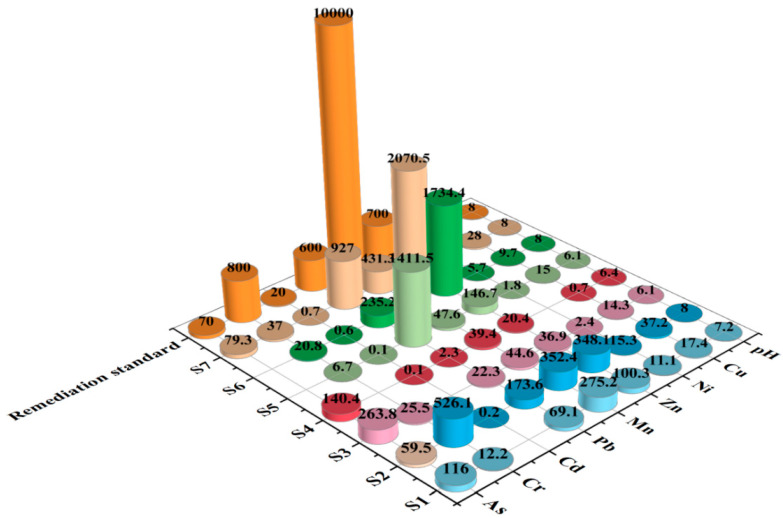
PMT content (mg·kg^−1^) and pH distribution in sampling sites inside a decommissioned REE plant.

The pH and PMTs content in the REE plant are shown in Figure 3. The pH value ranged from 6.1 to 8.0, and PMTs in the soils surveyed also varied remarkably. According to the soil remediation standard for PTM-contaminated sites [37], the standard for pH, Cu, Mn, Cd, Cr was met, while As, Zn, and Pb significantly exceeded the remediation standard. For instance, the highest As level of 263.8 mg·kg^−1^ was observed in S3, which was about four times the remediation standard value of 70 mg·kg^−1^ suggested by [37]. The highest Zn level of 2070.5 mg·kg^−1^ was measured in S7, which was nearly three times higher than the related standard value (700 mg·kg^−1^), while the highest Pb content of 1411.5 mg·kg^−1^ detected in S5 was over two times higher than the remediation standard (600 mg·kg^−1^). Concerning elemental correlationship (Figure 2b), most of the correlation coefficients between PTMs were positively correlated, such as Ni and Mn (0.99) and Zn and Cr (0.99). However, As was negatively correlated with other elements; the reason was not clear, but it may be ascribed to the underestimated content of As under the high drying and digestion temperatures in the sample treatment procedure. Generally, REE mineral extraction, refining, and related processing operations are the main sources of PTMs in the ambient soils, even though other artificial factors such as vehicle emissions inside or near the REE plant might also exerted some limited effects on the PTM content in soils.

The calculation results of single factor pollution index (*P*_m_) were as follows: As (9.4) > Zn (9.0) > Pb (8.0) > Cr (2.4) > Cd (2.3) > Cu (0.8) > Ni (0.6) > Mn (0.5). The results showed that As, Zn, and Pb were heavily polluted, while Cr and Cd were in the light pollution; Cu, Ni, and Mn did not reach contamination level. Special attention should be paid to Cu because it was almost close to threshold pollution value. Overall, these results were generally well consistent with the remediation standard assessment. With regard to comprehensive pollution index, the calculation result was 7.2, indicating that the PTMs of soils investigated were heavily polluted as a whole.

XRD analysis was performed to clarify the mineral composition of selected soil samples (S1, S2, S5, S6, and S7) for identification of the influences from rare earth element processing. As shown in Table 5, the major mineral phases of soil samples (S1, S2, S5) were mainly composed of quartz and kaolinite. It is important to note that S6 and S7 contained many minerals such as cuprite, arsenic phosphorus oxide, quenselite, copper silicon selenide, and cerussite that carried a great amount of PTMs like As, Pb, and Cu. Because these two soil sampling sites were largely different from others, they might be closely influenced by REE processing wastes, which can also in turn explain their relatively low quartz content. These XRD characterizations were generally well consistent with the PTMs distribution feature of Figure 3. Since the soil samples generally harbored identical or similar mineral assemblages with those present in the waste residues generated from REE processing, which was previously reported by Liu et al. [38], it can be safely concluded that the studied decommissioned REE plant had resulted in considerable PTM pollution after a decade of REE processing.

**Figure 4:**
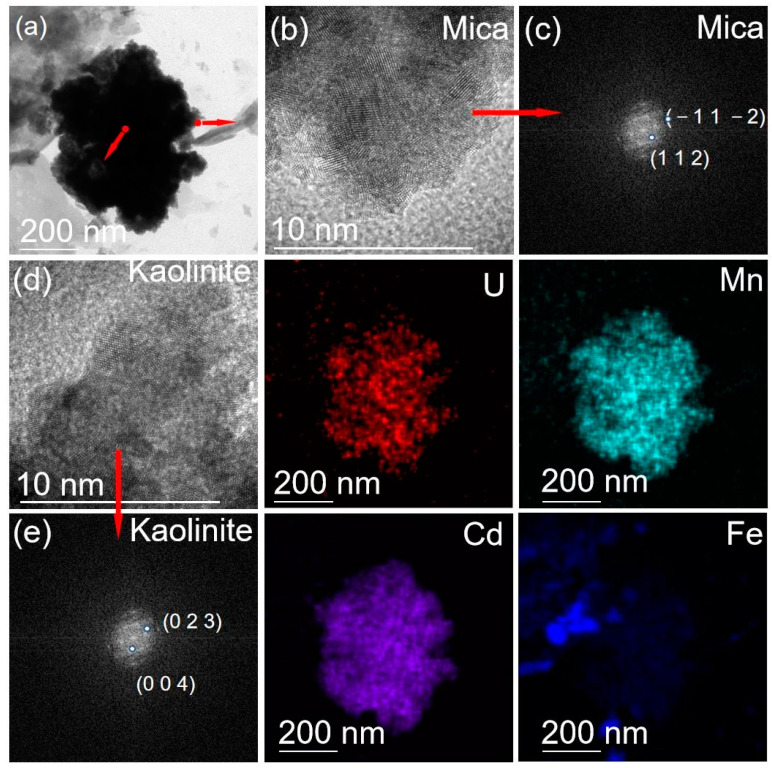
TEM mappings of selected S2 soil sample. (**a**) TEM image; (**b**,**d**) HRTEM image; (**c**,**e**) FFT image EM maps of particle.

**Figure 5:**
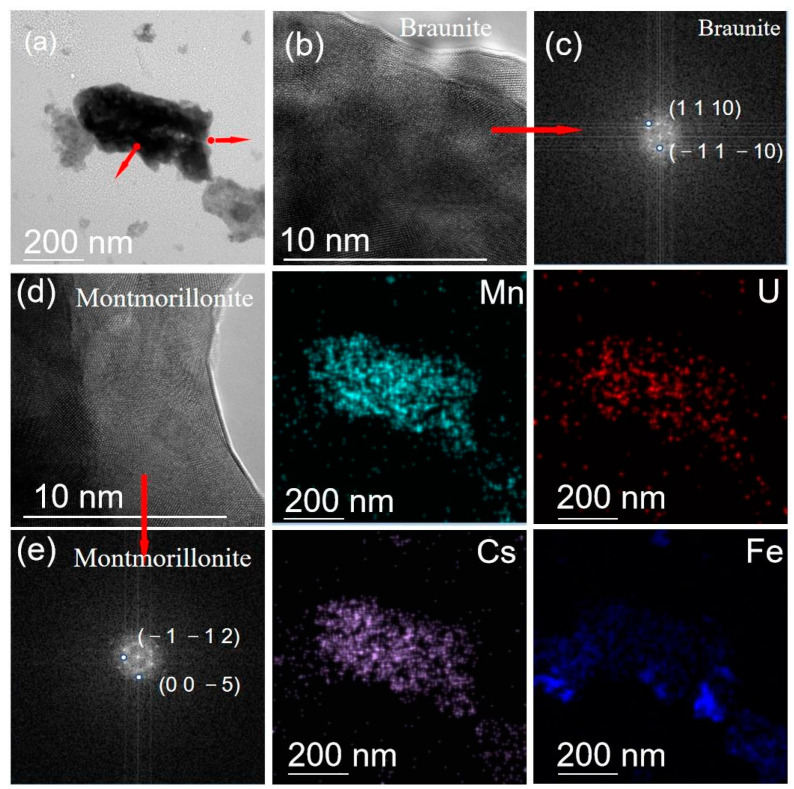
TEM mappings of selected S5 soil sample. (**a**) TEM image; (**b**,**d**) HRTEM image; (**c**,**e**) FFT image EM maps of particle.

The TEM analysis revealed the ultra-fine particles (<2 µm) from the soil samples of S2 and S5. According to the HRTEM images (Figure 4), fine particles of both kaolinite (Al_4_(OH)_8_(Si_4_O_10_)) and mica (KAl_2_(AlSi_3_O_10_)(OH)_2_) were observed in the soils, as generally reflected consistently in Table 5. Montmorillonite and braunite were also found in S5 (Figure 5). It is worth highlighting that visible U was commonly detected in elemental mappings, which was observed in both the mineral particles of mica and braunite, indicating the abundance of U in the samples agreed well with the above-mentioned radiological analysis results.

It is well known that U can gradually be accumulated in or adsorbed to clay minerals such as mica, kaolinite and montmorillonite [39,40]. Meanwhile, clay minerals can strongly adsorb PTMs, leading to excessive PTM content in soils. These results imply that more than expected areas of soils might have been seriously affected by REE processing activities.

## 4. Conclusions, Study Limitation and Perspective

The external radiation intensity of γ radiation and the specific activity of radioactive nuclides in the soil samples surveyed from a decommissioned rare earth element (REE) plant significantly exceeded the worldwide average values, posing a non-negligible health risk. Heavy pollution of potentially toxic metal(loid)s (PTMs) of arsenic, zinc and lead was observed in soil samples. It was assumed that more areas in REE plant might exceed the remediation standard in terms of total content of PTMs. Analyses by XRD and TEM characterizations of ambient soils further confirmed that high portion of elevated PTMs was stemming from the REE processing. In addition, clay minerals of montmorillonite and kaolinite were found to act as the major U hosting minerals in the soils. The preliminary study on radioactive nuclides and PTMs in the soils inside the REE plant is essential for predicting element migration and developing effective in situ remediation strategies.

On the other hand, the number of surveyed soils is limited, which constricts the width and depth of the present study; in addition, the PTM chemical speciation instead of total concentration analysis was not carried out. However, the fundamental data on radionuclides, air-absorbed dose rate of gamma radiation, as well as the PTM information were comprehensively presented to provide important reference for REE-induced environmental issues. Large-scale sampling with more profound physicochemical characterizations such as organic matter, grain size of the soils inside and outside the REE plant should be enhanced in the future.

## Figures and Tables

**Table 1:** The limits of detection (LOD) and validation data for target elements.

Method Code	ME-MS61r	ME-MS61r	ME-MS61r	ME-MS61r	ME-MS61r	ME-MS61r	ME-MS61r	ME-MS61r
Elements	Cu	Ni	Zn	Mn	Pb	Cd	Cr	As
Units	μg/g	μg/g	μg/g	μg/g	μg/g	μg/g	μg/g	μg/g
LOD	0.2	0.2	2	5	0.5	0.02	1	0.2
OREAS-45h	766	425	37	379	12.1	<0.02	612	18.4
OREAS-45h	803	449	40	391	11.7	<0.02	644	18.5
OREAS-45h	805	447	40	397	11.6	<0.02	647	17.8
Reference value	767	423	40	380	11.9	0.01	602	16.9
Blank	<0.2	<0.2	<2	<5	<0.5	<0.02	<1	<0.2

**Table 2:** The activity concentrations of ^238^U, ^226^Ra, ^232^Th, and ^40^K in surveyed soil samples in decommissioned REE processing plant (Bq·kg^−1^).

Sample	^238^U	^226^Ra	^232^Th	^40^K
S1	328.2	236.6	277.7	968.5
S2	1990.2	3767.2	2936.9	2157.6
S3	80.8	68.2	78.4	1020.8
S4	121.6	6196.2	8582.6	1045.3
S5	654.4	6935.0	14,372.4	2698.4
S6	182.1	99.6	151.1	625.4
S7	275.1	670.8	343.7	856.2
Range	80.8–1990.2	68.2–6935.0	78.4–8582.6	625.4–2698.4
Arithmetic mean	518.9	2567.7	3820.4	1338.9
Worldwide average *	40	35	30	400

* Data are abstracted from Singh et al. [24].

**Table 3:** Measured results of air-absorbed dose rate of γ radiation (nGy·h^−1^).

Sampling Point	Site Properties	Measured Values		
		Range	Average	Reference
S1	REE plant	149–163	155	present study
S2		780–860	826	present study
S3		221–251	231	present study
S4		371–4004	1610	present study
S5		2020–2231	2122	present study
S6		280–331	305	present study
S7		301–383	347	present study
Enugu state, Nigeria	Outdoor environment of urban areas	77.21–180.53	124.92	[29]
Ifonyintedo, Nigeria	Kaolin mining field	NG	59.6	[30]
Gauteng, South Africa	Gold mine tailings	NG	407.1	[31]
Gebeng, Kuantan	REE plant	29–118	65	[32]
Baotou and Bayan Obo, China	REE tailings dams	120–283	150	[33]
Moro, North-Central Nigeria	Gold and lead mining area	56.30–90.30	77.11	[34]
Binh Thuan, Vietnam	Coal-fired power plant	91.47–155.81	130.24	[35]
Worldwide average	Outdoor environment	NG	59	[36]

Note: NG not given.

**Table 4:** The calculated radium equivalent activity *R*_activity_, external radiation index *I*_r_, and annual effective dose rate *D*_aedr_ for sampling sites inside the decommissioned REE plant.

Sampling Site	*R*_activity_/Bq·kg^−1^	*D*_acdr_/μSv·a^−1^	*I*_r_
S1	708.3	190.1	1.91
S2	8133.1	1013.0	21.97
S3	258.9	283.3	0.70
S4	18549.8	1974.5	50.10
S5	27,695.3	2602.4	74.80
S6	363.8	374.1	0.98
S7	1228.2	425.6	3.32

**Table 5:** XRD-based mineralogy of soils sampled at different sites inside the decommissioned REE plant.

Mineral	Chemical Formula	S1	S2	S5	S6	S7
Quartz	SiO_2_	****	****	*****	**	**
Kaolinite	Al_4_(OH)_8_(Si_4_O_10_)	*****	*****			
Norsethite	BaMg(CO_3_)_2_					*
Calcite	CaCO_3_					****
Nepheline	KNa_3_(AlSiO_4_)_2_					****
Cuprite	Cu_2_O					****
Halite	NaCl				****	
Arsenic phosphorus oxide	AsPO_5_				****	
Quenselite	PbMnO_2_OH				**	
Copper silicon selenide	Cu_8_SiSe_6_				****	
Cerussite	PbCO_3_				**	
Lead arsenate	Pb(As_2_O_6_)			****		

Note: ***** major component (>50%); **** (20–50%); ** (5–10%); * (1–5%).

## Data Availability

All the research data have been included in the manuscript, others if any, can be available from the corresponding author upon reasonable request.
